# Foods contributing to nutrients intake and assessment of nutritional status in pre-dialysis patients: a cross-sectional study

**DOI:** 10.1186/s12882-020-01958-8

**Published:** 2020-07-25

**Authors:** Yeon Kyung Seo, Hyesu Lee, Hyunsuk Kim, Tae Yeon Kim, Hyunjin Ryu, Dal Lae Ju, Miyoung Jang, Kook-Hwan Oh, Curie Ahn, Sung Nim Han

**Affiliations:** 1 Department of Food and Nutrition, College of Human Ecology, Seoul National University, 1 Gwanak-ro, Gwanak-gu, Seoul, 08826 Korea; 2 Department of Internal Medicine, Chuncheon Sacred Heart Hospital, Hallym University Medical Center, Chuncheon-si, Gangwon-do Korea; 3 Department of Internal Medicine, Seoul National University College of Medicine, Seoul, Korea; 4 Department of Nutrition, SMG-SNU Boramae Medical Center, Seoul, Korea; 5 Department of Food Service and Nutrition Care, Seoul National University Hospital, Seoul, Korea; 6 Research Institute of Human Ecology, College of Human Ecology, Seoul National University, Seoul, Korea

**Keywords:** Pre-dialysis, Chronic kidney disease, Diabetes mellitus, Food intake, DII (dietary inflammatory index), BIA (bioelectrical impedance analysis), SGA (subjective global assessment)

## Abstract

**Background:**

For chronic kidney disease (CKD) patients, management of nutritional status is critical for delaying progression to end-stage renal disease. The purpose of this study is to provide the basis for personalized nutritional intervention in pre-dialysis patients by comparing the foods contributing to nutrients intake, nutritional status and potential dietary inflammation of CKD patients according to the diabetes mellitus (DM) comorbidity and CKD stage.

**Methods:**

Two hundred fifty-six outpatients referred to the Department of Nephrology at SNUH from Feb 2016 to Jan 2017 were included. Subjects on dialysis and those who had undergone kidney transplantation were excluded. Bioelectrical impedance analysis (BIA), subjective global assessment (SGA), dietary intake, and biochemical parameters were collected. Subjects were classified into 4 groups according to DM comorbidity (DM or Non-DM) and CKD stage (Early or Late) by kidney function. Two-way analysis of variance and multinomial logistic regression analysis were performed for statistical analysis.

**Results:**

Total number of malnourished patients was 31 (12.1%), and all of them were moderately malnourished according to SGA. The body mass index (BMI) of the DM-CKD group was significantly higher than the Non-DM-CKD group. The contribution of whole grains and legumes to protein intake in the DM-CKD group was greater than that in the Non-DM-CKD group. The DM- Early-CKD group consumed more whole grains and legumes compared with the Non-DM-Early-CKD group. The subjects in the lowest tertile for protein intake had lower phase angle, SGA score and serum albumin levels than those in the highest tertile. The potential for diet-induced inflammation did not differ among the groups.

**Conclusions:**

Significant differences in intakes of whole grains and legumes between CKD patients with or without DM were observed. Since contribution of whole grains and legumes to phosphorus and potassium intake were significant, advice regarding whole grains and legumes may be needed in DM-CKD patients if phosphorus and potassium intake levels should be controlled. The nutritional status determined by BIA, SGA and serum albumin was found to be different depending on the protein intake. Understanding the characteristics of food sources can provide a basis for individualized nutritional intervention for CKD patients depending on the presence of diabetes.

## Background

Chronic kidney disease (CKD) is a global health issue due to its poor clinical prognosis and high health care costs. Declining of estimated glomerular filtration rate (eGFR), an indication of kidney function, is associated with increased risk of cardiovascular disease (CVD) and death, as well as a longer duration of hospitalization [1]. For CKD patients with diabetes, controlling blood glucose levels and maintaining optimal nutritional status are critical for the prevention of progression to the end stage renal disease (ESRD) because diabetes mellitus (DM) can accelerate the progression of CKD [2].

Nutritional status is a major factor that affects the mortality [3] and quality of life in pre-dialysis patients [4]. Bioelectrical impedance analysis (BIA) and subjective global assessment (SGA) are used as screening tools to assess the nutritional status of CKD patients. Protein-energy wasting assessed by SGA was associated with increasing risk of death [5]. Malnutrition is a common condition in dialysis patients and DM comorbidity affects protein-energy wasting status [6]. In hemodialysis patients, phase angle (PhA), body mass index (BMI), and percent body fat measured by BIA were related to nutritional status according to the SGA classification [7]. Excess extracellular fluid (ECF) is common and can be a predictor of CVD morbidity in ESRD patients [8, 9].

The goal of treatment for CKD mainly aims to preserve kidney function, as kidney function continues to decrease with age and disease progression. Diet plays an important role in controlling proteinuria, which also reflects kidney function and nutritional status in CKD patients [10]. Compared to standard nutrition management, individualized medical nutrition therapy can improve the quality of life for CKD patients in the stage prior to ESRD [4]. KDIGO (Kidney Disease Improving Global Outcomes) guidelines recommend that CKD patients limit their protein and sodium intake as well as phosphorus and potassium intake if necessary [11]. Previous studies have focused on the nutrient intake levels of CKD patients and evaluated whether patients consume more than the levels recommended in the guidelines for CKD patients. Identifying the major food sources contributing to energy and nutrients intakes can help establish effective and practical strategies for nutritional care of patients. Among the nutrients, protein intake has a significant impact on kidney function and the type and quality of protein sources as well as its amount are important factors. In patients with eGFR < 60 mL/min/1.73 m^2^, a diet with a higher proportion of protein from plant sources was associated with lower all-cause mortality [12].

CKD involves low-grade inflammation. As CKD progresses to the ESRD, the inflammatory response increases due to elevated endotoxemia caused by oxidative stress and volume overload [13]. Several studies have shown that diet may be related to inflammation markers. According to Kaluza et al. [14], anti-inflammatory diet index can act as a predictor for low-grade systemic chronic inflammation. Dietary inflammatory index (DII) is used to assess the overall inflammatory potential of the diet and higher scores tend to indicate more pro-inflammatory diets. Rouhani et al. [15] reported that the tendency of a diet being pro-inflammatory was associated with disease progression in CKD patients. The potential dietary inflammation in pre-dialysis CKD patients with DM comorbidity has not been investigated.

Research that has focused on the food sources contributing to nutrients intake, comparison of intake of different food groups, and nutritional assessment in pre-dialysis patients is limited. The purpose of this study is to understand the differences among CKD groups by comparing food sources for nutrients, nutritional status and potential dietary inflammation according to DM comorbidity and CKD disease stage in order to provide the basis for better nutritional care of pre-dialysis CKD patients.

## Methods

### Study population

Initially 350 CKD patients referred to the Department of Nephrology at Seoul National University Hospital (SNUH) from Feb 2016 to Jan 2017 were recruited for the study. Patients who received kidney transplantation or were on dialysis were excluded from the study at the recruitment stage. Patients who had heart failure, chronic liver disease, and active cancer were also excluded from the study. Among the initial 350 subjects (159 DM-CKD and 191 Non-DM-CKD subjects), 94 subjects who did not complete 3-day dietary records were excluded. Therefore, final subjects included in the analysis were 256 CKD patients (107 DM-CKD and 149 Non-DM-CKD subjects). Subjects were classified based on two criteria: two groups based on DM comorbidity (DM-CKD and Non-DM-CKD groups); and two groups based on eGFR (Early-CKD and Late-CKD groups). All subjects signed a written informed consent before participation, and this study was approved by the Institutional Review Board of the SNUH (IRB No. H-1407-083-594).

### Demographic, clinical, anthropometric and biochemical data collection

The general characteristics of the subjects were collected using medical records (age, sex, comorbidity of diabetes mellitus and medication). SGA, BIA, anthropometric and biochemical measurements were done on the day when 3-day dietary records were collected from subjects. Weight and height were measured using an automatic measuring instrument (G-Tech International, Uijeongbu-si, Gyeonggi-do, Korea). Blood pressure was measured using fully automatic blood pressure monitors (A&D Company, Toshima-ku, Tokyo, Japan) after subjects were rested for more than 10 min. The following biochemical parameters were measured: blood urea nitrogen (BUN) (Urease/GLDH method), creatinine (Jaffe, Picrate/kinetic), albumin (BCG method) and fasting blood glucose (Hexokinase method).

The eGFR was calculated using CKD-EPI (Chronic Kidney Disease Epidemiology Collaboration) 2009 equation for all subjects [16] and classified into six stages based on the KDIGO guidelines; stage 1: 90 mL/min/1.73 m^2^ ≤ eGFR, stage 2: 60 ≤ eGFR < 90 mL/min/1.73 m^2^, stage 3a: 45 ≤ eGFR < 60 mL/min/1.73 m^2^, stage 3b: 30 ≤ eGFR < 45 mL/min/1.73 m^2^, stage 4: 15 ≤ eGFR < 30 mL/min/1.73 m^2^, stage 5: eGFR < 15 mL/min/1.73 m^2^ [11]. The Early-CKD group included the G1, G2 and G3a stages, and the Late-CKD group included the G3b, G4 and G5 stages in this study.

### BIA and SGA assessment for nutritional status

Inbody S10 device (Inbody, Seoul, Korea) was used for measuring body composition. The BIA data included weight, fat mass, fat free mass, skeletal muscle mass, percent body fat, visceral fat area, body mass index, phase angle, intracellular water, extracellular water, total body water, body cell mass and bone mineral content. A total of 30 impedances were measured using 6 frequencies (1, 5, 50, 250, 500, 1000 kHz) at 5 body segments (left and right arms, trunk, left and right legs).

Well-trained dietitian carried out the 7-point SGA using a 7-point Likert scale for the subjective ratings to assess nutritional status based on the medical history and physical examination. The medical history consisted of five criteria that focused on weight loss during 6 months, dietary intake change, gastrointestinal symptoms, functional capacity and co-morbidities that affect nutritional requirements based on the statement from patients. The physical examination evaluated subcutaneous fat, muscle wasting, ankle edema and/or ascites. Finally, the patients were classified into three SGA categories according to the score of each part and the general condition: A = well nourished (score 6 or 7), B = moderately malnourished (score 3, 4, or 5), C = severely malnourished (score 1 or 2).

### Assessment of dietary intake

Dietary intake was collected using 3-day dietary records. Food photograph booklets were provided to help subjects estimate portion sizes. Dietitians provided the instruction on how to write dietary records. Subjects recorded all foods and beverages consumed in two weekdays and one weekend day. Food and nutrient intake from the 3-day dietary records were calculated using the Computer Aided Nutritional Analysis Program (CAN-pro) version 5.0 (The Korean Nutrition Society, Seoul, Korea). None of the subjects were excluded from the analyses because no one was outside the predefined boundaries of total energy intake (< 800 kcal/day or > 4200 kcal/day for males; < 500 kcal/day or > 3500 kcal/day for females), limits recommended by Willet [17].

The major food sources contributing to energy, protein, potassium, phosphorus and sodium intakes were listed in descending order starting from the highest contributor. The percentage intake for each food of the total intake of each nutrient was calculated as follows: (intake of nutrients per food/total intake of nutrients per day of total nutrients) × 100. We identified the top 10 foods that contribute the most to the intake of energy and four nutrients.

### Analysis of dietary inflammatory index

DII scores were calculated using the method reported by Shivappa et al. [18] This study included 32 parameters of total 45 DII food and nutrients parameters as follows: energy, carbohydrate, protein, total fat, saturated fat, monounsaturated fatty acid, polyunsaturated fatty acid, n-3 fatty acid, n-6 fatty acid, cholesterol, fiber, vitamin A, C, D, E, B_6_, B_12_, β-carotene, thiamin, niacin, riboflavin, folic acid, iron, magnesium, selenium, zinc, ethanol, garlic, ginger, onion, green/black tea and pepper. Nutrient intake was adjusted by total energy intake using the residual method.

### Statistical analysis

All statistical analyses were performed using SAS, version 9.4 (SAS Institute, Cary, NC, USA). The Kolmogorov-Smirnov test was performed as the normality test for all variables. For the variables that do not follow a normal distribution, logarithmic or square-root transformation was performed. Demographic, clinical, anthropometric, biochemical and BIA data were analyzed using the chi-square test for categorical variables, and two-way ANOVA (Analysis of variance) for continuous variables. To compare the mean percent of food for protein, one-way ANOVA after logarithmic or square-root transformation was used, or the Kruskal-Wallis test if the variables did not meet normality. After one-way ANOVA to compare mean percentages among all groups, food that was significantly differentiated by group was analyzed by independent t-test according to DM comorbidity or GFR category.

Multinomial logistic regression analysis was used to evaluate the OR (odds ratios) and 95% CIs (confidence intervals) of 17 foods intake and DII for CKD patients according to DM comorbidity and GFR category. The Non-DM-Early-CKD group was used as the reference group. Trend tests for food intake in CKD patients were performed for significance by treating the median value of each tertile of food intake as a continuous variable. The multivariate model of foods intake according to the protein intake levels was adjusted for age (year, continuous), energy intake (kcal/day, continuous) and sex (male, female). Trend tests for the DII in CKD patients were performed for significance by treating the median value of each quartile of the DII as a continuous variable. The multivariate model of DII was adjusted for  age (year, continuous), energy intake (kcal/day, continuous), BMI (< 23, 23 < to < 25, > 25 kg/m^2^) and use of antihypertensive drug (yes, no). Pearson’s correlation coefficient was used to describe the correlation between two continuous variables. Statistical significance was set at a *P-*value < 0.05.

## Results

### Characteristics of subjects

The overall characteristics of the subjects are summarized in Table 1. The age of DM-CKD group was higher than Non-DM-CKD group (*P* < 0.01) and the age of Late-CKD group was higher than Early-CKD group (*P* < 0.01). Thirty-one moderately malnourished subjects were identified using SGA. The percentage of malnourished patients differed among the groups, with the lowest percentage in the Non-DM-Early-CKD group and the highest percentage in the DM-Late-CKD group. The percentage of subjects using anti-hypertensive medication differed among four groups (*P* < 0.05), and medication use was lowest in the Non-DM-Late-CKD group. Systolic blood pressure (SBP) of DM-CKD group was higher than that of Non-DM-CKD group (*P* < 0.01). Mean serum glucose level of the DM-CKD group was higher than the Non-DM-CKD group (*P* < 0.001). Mean serum albumin levels were within normal range. DM-Late-CKD group had lower serum albumin level than the DM-Early-CKD group while there was no difference between Non-DM -Early and Non-DM-Late-CKD groups.

**Table 1:** Characteristics of CKD patients according to DM comorbidity and GFR category

Parameters	Total (*n* = 256)	DM-CKD (*n* = 107)		Non-DM-CKD (*n* = 149)		*P* value^3,4^		
		Early-CKD (*n* = 49)	Late-CKD (*n* = 58)	Early-CKD (*n* = 77)	Late-CKD (*n* = 72)	DM comorbidity	GFR category	Interaction
**Sociodemographic parameter**								
Age (years)	59.9 ± 12.1^1^	60.7 ± 11.3^a^	63.4 ± 11.0^a^	56.0 ± 12.0^b^	60.5 ± 12.7^a^	< 0.01	< 0.01	1.00
**Medication and supplements**								
Antihypertensive drug use, n (%)	223 (87.1)	44 (89.8)	53 (91.4)	70 (90.9)	56 (77.8)		< 0.05	
**Clinical parameters**								
eGFR (mL/min/1.73m^2^)	50.4 ± 27.3^1^	71.4 ± 20.0^a^	29.0 ± 8.9^b^	74.6 ± 19.6^a^	27.5 ± 10.0^b^	0.08	< 0.001	1.00
SBP (mmHg)^5^	128.1 ± 12.7	129.3 ± 9.5^ab^	132.4 ± 15.5^a^	124.9 ± 12.0^b^	127.1 ± 11.8^ab^	< 0.01	0.07	1.00
DBP (mmHg)^5^	77.8 ± 9.9	78.2 ± 9.9	76.7 ± 10.2	78.0 ± 10.6	78.1 ± 9.4	0.63	0.52	0.66
Malnutrition, n (%)^6^	31 (12.1)^2^	6 (12.2)	12 (20.7)	1 (1.3)	12 (16.7)		< 0.01	
**Biochemical data**								
Creatinine (mg/dL)								
Male	1.83 ± 0.88	1.2 ± 0.3^b^	2.3 ± 0.7^a^	1.3 ± 0.2^b^	2.6 ± 1.0^a^	0.70	< 0.001	0.15
Female	1.64 ± 1.02	0.8 ± 0.2^b^	2.4 ± 1.0^a^	0.8 ± 0.2^b^	2.4 ± 1.0^a^	0.96	< 0.001	0.53
BUN (mg/dL)	26.9 ± 15.3	18.5 ± 6.8^b^	35.0 ± 17.3^a^	16.3 ± 4.3^b^	37.2 ± 14.8^a^	0.24	< 0.001	0.08
Glucose (mg/dL)	114.5 ± 30.7	127.3 ± 28.7^a^	138.0 ± 45.6^a^	101.6 ± 10.4^b^	100.6 ± 9.7^b^	< 0.001	0.09	0.21
Serum albumin (g/dL)	4.2 ± 0.6	4.3 ± 0.3^a^	4.1 ± 0.4^c^	4.2 ± 0.3^ab^	4.2 ± 0.3^b^	0.69	< 0.001	< 0.01

^1^The data are presented as means ± standard deviations

^2^The data are presented as number (percentage)

^3^Two-way ANOVA was performed to compare parameters according to the DM comorbidity and GFR category. Means with different letters indicate significant differences (*P* < 0.05) by Bonferroni multiple comparison test

^4^Chi-square test was performed to compare the categorical parameters according to the DM comorbidity and GFR category

^5^SBP and DBP data were missing in 8 subjects

^6^Malnutrition was defined using SGA by the clinical dietitian

The anthropometric characteristics and BIA data of the subjects are shown in Table 2. BMI was higher in the DM-CKD group than in the Non-DM-CKD group (men *P* = 0.04; women *P* < 0.01). Among women, weight (*P* < 0.01) and skeletal muscle mass (*P* = 0.01) was higher in DM-CKD group than in Non-DM-CKD group. In men, percent of body fat (PBF) and visceral fat area (VFA) were higher in DM-CKD group than in Non-DM-CKD group (PBF *P* = 0.04; VFA *P* < 0.01). Fat mass of men was higher in Early-CKD group than in Late-CKD group (*P* = 0.04).

**Table 2:** Anthropometric characteristics and BIA measurement of the subjects according to DM comorbidity and GFR category

Parameters	Total (*n* = 256)	DM-CKD (*n* = 107)		Non-DM-CKD (*n* = 149)		*P* value^3^		
		Early-CKD (*n* = 49)	Late-CKD (*n* = 58)	Early-CKD (*n* = 77)	Late-CKD (*n* = 72)	DM comorbidity	GFR category	Interaction
**Anthropometric parameters**								
Height (cm)								
Male	168.4 ± 6.1^1^	166.9 ± 6.2	168.4 ± 6.4	168.6 ± 6.2	169.5 ± 5.5	0.20	0.30	0.51
Female	155.5 ± 5.7	157.5 ± 3.6	153.8 ± 6.8	156.3 ± 4.6	154.7 ± 6.4	0.91	0.03	0.38
**Bioelectrical impedance analysis(BIA)**								
Weight (kg)								
Male	71.4 ± 11.5	73.2 ± 14.8	72.2 ± 11.0	71.8 ± 8.5	68.7 ± 12.1	0.23	0.31	0.43
Female	61.5 ± 10.9	65.8 ± 12.1^a^	64.8 ± 13.3^ab^	60.8 ± 8.6^ab^	57.9 ± 9.6^b^	< 0.01	0.28	0.73
Body mass index, BMI (kg/m^2^)								
Male	25.1 ± 3.3	26.1 ± 4.0	25.4 ± 3.2	25.2 ± 2.6	23.8 ± 3.5	0.04	0.07	0.25
Female	25.4 ± 4.2	26.4 ± 4.2^ab^	27.3 ± 5.0^a^	24.9 ± 3.7^ab^	24.2 ± 3.7^b^	< 0.01	0.85	0.34
Fat mass, FM (kg) ^2^								
Male	17.8 ± 7.0	19.8 ± 9.3	18.4 ± 7.1	17.9 ± 4.9	15.5 ± 6.8	0.11	0.04	0.18
Female	21.9 ± 7.4	23.3 ± 8.4	23.9 ± 9.2	21.1 ± 6.7	20.7 ± 6.2	0.10	0.88	0.84
Percent body fat, PBF (%)								
Male	24.5 ± 6.4	26.2 ± 6.8	25.3 ± 6.7	24.8 ± 5.2	22.0 ± 6.7	0.04	0.10	0.19
Female	35.0 ± 6.3	35.1 ± 5.8	36.2 ± 8.1	34.2 ± 6.1	35.1 ± 5.7	0.46	0.44	0.90
Fat free mass, FFM (kg)								
Male	53.5 ± 7.1	22.0 ± 6.7	53.8 ± 7.4	53.7 ± 6.5	53.1 ± 7.4	0.85	0.95	0.68
Female	39.4 ± 5.3	41.5 ± 5.1^a^	40.9 ± 7.1^ab^	39.7 ± 3.7^ab^	37.2 ± 4.7^b^	< 0.01	0.07	0.41
Skeletal muscle mass, SMM (kg)								
Male	29.6 ± 4.3	29.7 ± 4.4	29.4 ± 4.6	30.0 ± 3.9	29.4 ± 4.5	0.79	0.51	0.81
Female	21.2 ± 3.2	22.4 ± 3.0^a^	21.9 ± 4.1^ab^	21.4 ± 2.3^ab^	19.9 ± 2.9^b^	0.01	0.07	0.49
Visceral fat area, VFA (cm^2^) ^2^								
Male	77.1 ± 31.8	86.1 ± 40.0	83.6 ± 33.4	74.3 ± 22.3	66.6 ± 30.3	< 0.01	0.21	0.13
Female	105.1 ± 39.5	112.8 ± 39.4	115.9 ± 46.9	97.9 ± 38.1	101.0 ± 35.6	0.09	0.76	0.84
Midarm circumference, MAC (cm) ^2^								
Male	26.0 ± 2.4	25.8 ± 4.0	25.8 ± 1.8	26.5 ± 1.8	25.7 ± 2.1	0.33	0.66	0.28
Female	23.2 ± 2.0	23.9 ± 1.9	23.9 ± 2.6	23.1 ± 1.6	22.5 ± 1.9	< 0.01	0.41	0.50
Phase angle, PhA (degrees)	5.3 ± 0.8	5.3 ± 0.7^b^	5.0 ± 0.7^b^	5.7 ± 0.7^a^	5.2 ± 0.8^b^	< 0.001	< 0.001	1.00
Body cell mass, BCM (kg)	31.0 ± 6.2	31.8 ± 5.8	31.7 ± 6.2	31.4 ± 5.9	29.4 ± 6.7	0.10	0.12	0.19
Intracellular water, ICW (L)	21.6 ± 4.4	22.2 ± 4.0	22.1 ± 4.4	22.0 ± 4.1	20.5 ± 4.7	0.10	0.13	0.18
Extracellular water, ECW (L)	13.6 ± 2.8	14.0 ± 2.5^ab^	14.3 ± 2.8^a^	13.3 ± 2.7^ab^	12.9 ± 2.8^b^	< 0.01	0.73	0.27
Total body water, TBW (L)	35.0 ± 7.4	36.2 ± 6.5	36.4 ± 7.1	35.2 ± 7.2	33.0 ± 8.1	0.02	0.24	0.17
Extracellular fluid, ECF (ECW/TBW)	0.39 ± 0.01	0.39 ± 0.01^a^	0.39 ± 0.01^b^	0.38 ± 0.01^c^	0.39 ± 0.01^a^	< 0.001	< 0.001	1.00
Bone mineral mass, BMC (kg)	2.6 ± 0.5	2.7 ± 0.5	2.7 ± 0.6	2.7 ± 0.5	2.5 ± 0.5	0.05	0.23	0.20

^1^The data are presented as means ± standard deviations

^2^The variable was analyzed after transformed to natural logarithms

^3^Two-way ANOVA was performed to compare parameters according to the DM comorbidity and GFR category. Means with different letters indicate significant differences (*P* < 0.05) by Bonferroni multiple comparison test

Mean energy and protein intakes are presented in Table 3. Subjects in the Late-CKD group consumed less energy and protein than those in the Early-CKD group in this study (both men and women). Average protein intake per day was significantly lower in the Late-CKD group compared with the Early-CKD group. Non-DM-CKD group tended to consume less protein than DM-CKD group in women (*P* = 0.06), but not in men.

**Table 3:** Mean energy and protein intake of subjects according to DM comorbidity and GFR category

Parameters	Total	DM-CKD		Non-DM-CKD		*P* value^2^		
		Early-CKD	Late-CKD	Early-CKD	Late-CKD	DM comorbidity	GFR category	Interaction
**Energy intake (kcal)**								
Male	1845.5 ± 441.1^1^	2004.2 ± 469.7	1756.5 ± 406.1	1902.4 ± 452.8	1731.8 ± 398.4	0.57	< 0.01	0.39
Female	1517.9 ± 415.7	1775.1 ± 297.9^a^	1356.2 ± 338.1^b^	1543.1 ± 459.6^ab^	1456.5 ± 418.4^ab^	0.48	< 0.01	< 0.05
**Protein intake (g)**								
Male	72.5 ± 23.3	84.4 ± 21.2^a^	67.2 ± 23.3^b^	73.7 ± 22.6^ab^	66.6 ± 22.9^b^	0.24	< 0.01	0.10
Female	59.0 ± 19.4	70.8 ± 18.9^a^	57.0 ± 16.2^ab^	59.9 ± 21.0^ab^	53.4 ± 17.7^b^	0.06	0.01	0.38

^1^The data are presented as means ± standard deviations

^2^Two-way ANOVA was performed to compare parameters according to the DM comorbidity and GFR category. Means with different letters indicate significant differences (*P* < 0.05) by Bonferroni multiple comparison test

### Percent contribution of the top 10 food sources for protein intake

The top 10 foods contributing to protein intake, in descending order, were refined grains, fish, red meat, legumes, eggs, whole grains, seafood, cooked vegetables, poultry and seasoning (Table 4). The percent contribution of refined grains (*P* < 0.01), legumes (*P* < 0.01) and whole grains (*P* < 0.01) for protein intake was different among four groups. The mean percentage contribution for protein intake by legumes and whole grains were higher in the DM-CKD group than the Non-DM-CKD group, while mean percentage contribution by refined grains was lower in the DM-CKD group than Non-DM-CKD group. Foods contributing to energy, phosphorus, potassium and sodium for all CKD patients, DM-CKD patients only, and Non-DM-CKD patients only are listed in Supplement tables 1, 2, and 3.

**Table 4:** Major foods contributing to protein intake of CKD patients according to DM comorbidity and GFR category

Ranking (% of Protein/d)	Total (*n* = 256)	DM-CKD (*n* = 107)		Non-DM-CKD (*n* = 149)		*P* value^2, 3, 4^
		Early-CKD (*n* = 49)	Late-CKD (*n* = 58)	Early-CKD (*n* = 77)	Late-CKD (*n* = 72)	
1 Refined grains^4,5^	17.1 ± 0.5^1^	14.6 ± 1.1^b^	16.5 ± 1.0^ab^	16.7 ± 1.9^ab^	19.7 ± 1.0^a^	< 0.01
2 Fish^4^	13.0 ± 0.7	11.7 ± 1.3	14.0 ± 1.4	13.2 ± 1.5	12.7 ± 1.4	0.76
3 Red meat^4^	12.9 ± 0.6	14.4 ± 1.3	12.0 ± 1.3	13.2 ± 1.5	12.5 ± 1.1	0.49
4 Legumes^4, 5^	8.5 ± 0.5	10.8 ± 1.2^a^	10.4 ± 1.4^ab^	6.7 ± 0.8^b^	7.2 ± 0.7^ab^	< 0.01
5 Eggs^4^	5.4 ± 0.3	5.0 ± 0.5	4.6 ± 0.6	5.5 ± 0.6	6.3 ± 0.7	0.37
6 Whole grains^4, 5^	4.9 ± 0.4	6.4 ± 0.7^a^	6.2 ± 1.0^ab^	3.9 ± 0.4^b^	4.0 ± 0.7^b^	< 0.01
7 Seafood^2^	4.5 ± 0.4	3.7 ± 0.8	4.0 ± 1.0	5.3 ± 0.6	4.7 ± 0.8	0.31
8 Cooked vegetables^2^	4.3 ± 0.2	4.2 ± 0.3	4.1 ± 0.4	4.3 ± 0.5	4.6 ± 0.4	0.58
9 Poultry^2^	4.2 ± 0.5	5.0 ± 1.2	5.1 ± 1.5	4.3 ± 0.5	2.7 ± 0.8	0.83
10 Seasoning^3^	3.5 ± 0.1	3.7 ± 0.3	3.5 ± 0.2	3.5 ± 0.4	3.5 ± 0.3	0.88

^1^The data are presented as means ± SEMs (standard error of means)

^2^The variable was analyzed after transformed to natural logarithms and one-way ANOVA was used

^3^The variable was analyzed after transformed to square root and one-way ANOVA was used

^4^Kruskal-Wallis test was used

^5^Means with different letters indicate significant differences (*P* < 0.05) by Bonferroni multiple comparison test

### Comparison of food intake

For protein intake from animal and plant sources, the tertile of each food group intake was compared among 4 groups (Tables 5 and 6). The reference group was the Non-DM-Early-CKD group. Among all selected foods examined in the multivariate models, consumption of whole grains (OR:3.92; 95% CIs:1.43–10.77; *P*_trend_ < 0.01) and legumes (OR:5.12; 95% CIs:1.74–15.07; *P*_trend_ < 0.01) was higher in the DM-Early-CKD group compared with the Non-DM-Early-CKD group. Salted vegetables intake (OR:0.40; 95% CIs:0.17–0.98; *P*_trend_ = 0.03) was lower in the Non-DM-Late- CKD group compared with the Non-DM-Early-CKD group. In the DM- Late-CKD group, consumption of seafood (OR:0.36; 95% CIs:0.15–0.88; *P*_trend_ = 0.03) and dairy (OR:0.39; 95% CIs:0.17–0.90; *P*_trend_ = 0.03) was lower compared with the Non-DM-Early-CKD group.

**Table 5:** Odds ratios (ORs) and 95% confidence intervals (CIs) of multivariate model for CKD patients according to tertiles of animal protein sources intake

	Tertile	Non-DM-Early-CKD^1^ (*n* = 77)	DM-Early-CKD (*n* = 49)		Non-DM-Late-CKD (*n* = 72)		DM-Late-CKD (*n* = 58)	
		Reference	Multivariate-adjusted model^2^ OR (95% CIs)	*P*_trend_^3^	Multivariate-adjusted model OR (95% CIs)	*P*_trend_	Multivariate-adjusted model OR (95% CIs)	*P*_trend_
**Red meat (g)**	< 23	1	1	0.26	1	0.80	1	0.65
	23–57	1	1.81		1.54		1.58	
			(0.67–4.89)		(0.68–3.47)		(0.66–3.79)	
	57<	1	1.91		1.22		1.36	
			(0.72–5.12)		(0.50–2.96)		(0.52–3.60)	
**Processed red meat (g)**	0	1	1	0.22	1	0.50	1	0.09
	0 < −15	1	1.60		2.61*		0.72	
			(0.59–4.31)		(1.10–6.37)		(0.24–2.21)	
	15<	1	0.53		0.68		0.41	
			(0.18–1.54)		(0.24–1.96)		(0.13–1.30)	
**Meat byproducts (g)**	0 (*n* = 226)	1	1	–	1	–	1	–
	0 < (*n* = 30)	1	0.42		0.65		0.50	
			(0.13–1.42)		(0.25–1.71)		(0.16–1.56)	
**Poultry (g)**	0	1	1	0.87	1	0.09	1	0.38
	0 < −40	1	0.74		0.56		0.78	
			(0.25–2.25)		(0.21–1.50)		(0.27–2.28)	
	40<	1	1.12		0.40		1.60	
			(0.42–2.97)		(0.13–1.24)		(0.61–4.23)	
**Eggs (g)**	< 15	1	1	0.73	1	0.72	1	0.46
	15–40	1	1.67		0.97		1.28	
			(0.65–4.30)		(0.42–2.24)		(0.54–3.05)	
	40+	1	0.97		0.88		0.75	
			(0.42–2.24)		(0.39–2.00)		(0.30–1.85)	
**Fish (g)**	< 13	1	1	0.55	1	0.45	1	0.89
	13–44	1	1.24		1.02		1.28	
			(0.50–3.08)		(0.45–2.29)		(0.53–3.10)	
	44<	1	0.81		0.75		1.00	
			(0.32–2.03)		(0.33–1.70)		(0.41–2.43)	
**Processed fish (g)**	0	1	1	0.85	1	0.65	1	0.91
	0 < −15	1	1.38		0.76		0.41	
			(0.53–3.60)		(0.29–2.00)		(0.12–1.39)	
	15<	1	1.08		1.27		1.03	
			(0.39–2.98)		(0.51–3.18)		(0.38–2.80)	
**Seafood (g)**	0	1	1	0.49	1	0.42	1	0.03
	0 < −16	1	0.94		0.57		0.52	
			(0.37–2.42)		(0.24–1.33)		(0.22–1.25)	
	16<	1	0.73		0.69		0.36*	
			(0.30–1.82)		(0.31–1.52)		(0.15–0.88)	
**Milk (ml)**	2<	1	1	0.42	1	0.42	1	0.64
	2–40	1	0.68		2.30		2.36	
			(0.11–4.07)		(0.61–8.63)		(0.58–9.64)	
	40<	1	0.71		0.77		0.87	
			(0.32–1.57)		(0.37–1.62)		(0.39–1.91)	
**Dairy (g)**	0<	1	1	0.14	1	0.88	1	0.03
	0–9	1	0.90		1.65		0.87	
			(0.25–3.23)		(0.52–5.22)		(0.24–3.12)	
	9<	1	0.55		1.03		0.39*	
			(0.24–1.23)		(0.50–2.11)		(0.17–0.90)	

*Significant at the 0.05 level

^1^The Non-DM-Early-CKD group was used as a reference

^2^Adjusted for age (years,  continuous), sex (male, female) and energy intake (kcal,  continuous)

^3^Based on logistic regression analysis with assignment of median values to each category

**Table 6:** Odds ratios (ORs) and 95% confidence intervals (CIs) of multivariate model for CKD patients according to tertiles of plant protein sources intake

	Tertile	Non-DM-Early- CKD^1^ (*n* = 77)	DM-Early-CKD (*n* = 49)		Non-DM-Late-CKD (*n* = 72)		DM-Late-CKD (*n* = 58)	
		Reference	Multivariate-adjusted model^2^ OR (95% CIs)	*P*_trend_^3^	Multivariate-adjusted model OR (95% CIs)	*P*_trend_	Multivariate-adjusted model OR (95% CIs)	*P*_trend_
**Refined grains (g)**	< 135	1	1	0.20	1	0.06	1	0.40
	135–207	1	0.44		2.34		3.00*	
			(0.16–1.18)		(0.98–5.56)		(1.21–7.46)	
	207<	1	0.53		2.53		1.61	
			(0.20–1.38)		(0.98–6.51)		(0.57–4.53)	
**Whole grains (g)**	< 4	1	1	< 0.01	1	0.91	1	0.25
	4–40	1	1.65		0.65		1.22	
			(0.59–4.61)		(0.30–1.43)		(0.51–2.88)	
	40<	1	3.92*		0.89		1.72	
			(1.43–10.77)		(0.38–2.09)		(0.69–4.31)	
**Legumes (g)**	< 18	1	1	< 0.01	1	0.83	1	0.30
	18–55	1	3.61*		1.30		1.43	
			(1.23–10.66)		(0.60–2.84)		(0.60–3.39)	
	55<	1	5.12*		1.15		1.66	
			(1.74–15.07)		(0.49–2.68)		(0.68–4.05)	
**Raw vegetables (g)**	< 23	1	1	0.45	1	0.66	1	0.93
	23–58	1	1.02		2.10		1.03	
			(0.40–2.59)		(0.92–4.76)		(0.43–2.46)	
	58<	1	1.35		1.43		0.97	
			(0.56–3.23)		(0.62–3.32)		(0.41–2.30)	
**Cooked vegetables (g)**	< 89	1	1	0.91	1	0.81	1	0.38
	89–139	1	0.87		0.62		0.64	
			(0.33–2.26)		(0.28–1.42)		(0.27–1.53)	
	139<	1	0.93		0.85		0.63	
			(0.34–2.51)		(0.36–2.02)		(0.25–1.62)	
**Salted vegetables (g)**	< 47	1	1	0.37	1	0.03	1	0.60
	47–94	1	2.07		0.97		0.84	
			(0.78–5.47)		(0.43–2.18)		(0.32–2.18)	
	94<	1	0.86		0.40*		1.2	
			(0.31–2.43)		(0.17–0.98)		(0.50–2.90)	
**Seeds and nuts (g)**	1<	1	1	0.54	1	0.28	1	0.80
	1–7	1	2.08		1.01		1.16	
			(0.81–5.33)		(0.45–2.27)		(0.47–2.85)	
	7<	1	1.16		0.68		1.18	
			(0.44–3.05)		(0.30–1.54)		(0.49–2.80)	

*Significant at the 0.05 level

^1^The Non-DM-Early-CKD group was used as a reference

^2^Adjusted for age (years,  continuous), sex (male, female) and energy intake (kcal,  continuous)

^3^Based on logistic regression analysis with assignment of median values to each category

### Characteristics of CKD patients across DII quartiles

The characteristics of subjects across DII quartiles are summarized in Table 7. DII scores ranged from − 3.77 to 5.46, with a median of 2.21. Compared to subjects in the lowest quartile (most anti-inflammatory DII category), those in the highest quartile (most pro-inflammatory) were significantly younger (*P* < 0.001). The number of malnourished subjects differed among the quartiles (*P* < 0.01), with the highest proportion of malnutrition in quartile 2. There was no difference in the proportion of CKD stage and DM comorbidity distribution among DII quartiles.

**Table 7:** Characteristics of the subjects according to quartiles of dietary inflammatory index (DII)

Characteristics	Quartiles of dietary inflammatory index (*n* = 256)				*P* value^3,4^
	Q1 (*n* = 64)	Q2 (*n* = 64)	Q3 (*n* = 64)	Q4 (*n* = 64)	
**DII**					
Median	0.53	1.71	2.67	3.75	
Minimum, maximum	−3.77, 1.14	1.19, 2.21	2.22, 3.13	3.13,5.46	
**Age**	64.1 ± 9.5^a^	58.3 ± 11.5^b^	61.5 ± 12.5^ab^	55.6 ± 13.1^b^	< 0.001
**Male (%)**	38 (59.4)^2^	34 (53.1)	40 (62.5)	41 (64.1)	0.6
**Weight (kg)**	67.2 ± 11.8^1^	64.6 ± 9.8	68.1 ± 12.8	69.8 ± 14.0	0.11
**BMI, kg/m**^**2**^	25.3 ± 3.8	24.4 ± 2.9	25.4 ± 3.8	25.9 ± 4.2	0.16
**CKD stage (%)**					0.38
Stage 1	6 (9.4)	6 (9.4)	8 (12.5)	7 (10.9)	
Stage 2	10 (15.6)	17 (26.6)	11 (17.2)	21 (32.8)	
Stage 3a	12 (18.8)	13 (20.3)	9 (14.1)	7 (10.9)	
Stage 3b	19 (29.7)	9 (14.1)	16 (25.0)	13 (20.3)	
Stage 4	15 (23.4)	16 (25.0)	15 (23.4)	10 (15.6)	
Stage 5	2 (3.1)	3 (4.7)	5 (7.8)	6 (9.4)	
**eGFR, mL/min/1.73m**^**2**^	47.6 ± 25.0	52.7 ± 26.4	48.9 ± 29.3	52.5 ± 28.8	0.63
**SBP, mmHg**^5^	129.7 ± 12.9	126.0 ± 13.2	128.3 ± 11.2	128.1 ± 13.4	0.46
**DBP, mmHg**^5^	77.5 ± 10.5	76.3 ± 10.6	77.6 ± 9.4	79.7 ± 9.3	0.29
**Current diabetes, n (%)**	31 (48.4)	25 (39.1)	27 (42.2)	24 (37.5)	0.61
**Fasting blood glucose, mg/dL**	120.5 ± 36.4	112.6 ± 35.2	115.1 ± 22.4	109.9 ± 26.4	0.25
**Malnutrition (%)**^6^	4 (6.3)	16 (25.0)	7 (10.9)	4 (6.3)	< 0.01

^1^The data are presented as means ± standard deviations

^2^The data are presented as number (percentage)

^3^One-way ANOVA was performed to compare the continuous parameters according to the DM comorbidity and GFR category

^4^Chi-square test was performed to compare the categorical parameters according to the DM cmorbidity and GFR category

^5^SBP and DBP data were missing in 8 subjects

^6^Malnutrition was defined using SGA by the clinical dietitian

### Comparison of methods for malnutrition assessments according to protein intake

Protein intake was divided into tertile or low-protein diet (protein intake below 0.8 g/kg/d) and BIA, SGA and serum albumin levels were compared according to the levels of protein intake (Tables 8 and 9). Patients in the third quartile of protein intake were found to have significantly higher phase angle (PhA), body cell mass (BCM), intracellular water (ICW), extracellular water (ECW), total body water (TBW), bone mineral content (BMC) and SGA score and lower extracellular fluid (ECF). The number of malnourished patients according to the SGA differed significantly among the tertiles, and the proportion of malnourished patients was the highest in the first tertile (the lowest protein intake). The SGA score did not differ between  the groups below and above 0.8 g/kg/day protein intake, but the proportion of malnourished was higher in those with protein intake below 0.8 g/kg/day. Supplement tables 4, 5, 6, 7 show the BIA results according to the protein intake levels in men and women.

**Table 8:** Bioelectrical impedance analysis (BIA) measurement, subjective global assessment (SGA) and serum albumin levels  of the all subjects according to tertiles of protein intake

Parameters	Tertiles of protein intake (*n* = 256)^1^			*P* value^3,6^
	Tertile 1 (*n* = 85)	Tertile 2 (*n* = 86)	Tertile 3 (*n* = 85)	
**Bioelectrical impedance analysis**				
Phase angle, PhA (degrees)	5.1 ± 0.7^b 2^	5.3 ± 0.7^b^	5.6 ± 0.8^a^	< 0.001
Body cell mass, BCM (kg)	29.3 ± 7.0^b^	30.9 ± 5.7^ab^	32.8 ± 5.5^a^	< 0.01
Intracellular water, ICW (L)	20.4 ± 4.9^b^	21.6 ± 4.0^ab^	22.9 ± 3.8^a^	< 0.01
Extracellular water, ECW (L)	13.0 ± 3.0^b^	13.6 ± 2.4^ab^	14.1 ± 2.7^a^	0.02
Total body water, TBW (L)	33.4 ± 7.9^b^	34.6 ± 7.8^ab^	37.1 ± 6.1^a^	< 0.01
Extracellular fluid, ECF (ECW/TBW)	0.39 ± 0.01^a^	0.39 ± 0.01^ab^	0.38 ± 0.01^b^	< 0.001
Bone mineral content, BMC (kg)	2.5 ± 0.6^b^	2.6 ± 0.4^ab^	2.8 ± 0.4^a^	< 0.01
**Subjective global assessment**				
SGA score	6.3 ± 0.9^b^	6.5 ± 0.7^a^	6.6 ± 0.7^a^	0.04
Malnutrition, n (%) ^5^	18 (21.2)^4^	7 (8.1)	6 (7.1)	< 0.01
**Biochemical data**				
Serum albumin (g/dL)	4.1 ± 0.3^b^	4.2 ± 0.3^ab^	4.3 ± 0.3^a^	< 0.01

^1^ The subjects were classified into 3 groups according to tertiles of protein intake in all subjects

^2^ The data are presented as means ± standard deviations

^3^ One-way ANOVA was performed to compare parameters according to tertiles of protein intake. Means with different letters indicate significant differences (*P* < 0.05) by Bonferroni multiple comparison test

^4^ The data are presented as number (percentage)

^5^ Malnourished patients were assessed using modified SGA (subjective global assessment)

^6^ Chi-square test was performed to compare the categorical parameters (malnutrition) according to tertiles of protein intake

**Table 9:** Bioelectrical impedance analysis (BIA) measurement, subjective global assessment (SGA) and serum albumin levels  of all subjects according to low protein diet (< 0.8 g/kg/day)

Parameters	All (*n* = 256)^1^		*P* value^3,6^
	Low-protein diet (*n* = 65)	Non-low-protein diet (*n* = 191)	
**Bioelectrical impedance analysis**			
Phase angle, PhA (degrees)	5.2 ± 0.7^2^	5.3 ± 0.8	0.38
Body cell mass, BCM (kg)	31.7 ± 7.0	30.7 ± 6.0	0.26
Intracellular water, ICW (L)	22.2 ± 4.9	21.5 ± 4.2	0.24
Extracellular water, ECW (L)	14.0 ± 3.0	13.4 ± 2.7	0.11
Total body water, TBW (L)	36.2 ± 7.8	34.6 ± 7.3	0.14
Extracellular fluid, ECF (ECW/TBW)	0.39 ± 0.01	0.39 ± 0.01	0.07
Bone mineral content, BMC (kg)	2.7 ± 0.6	2.6 ± 0.5	0.32
**Subjective global assessment**			
SGA score	6.4 ± 0.9	6.5 ± 0.7	0.46
Malnutrition, n (%)^5^	13 (20.0)^4^	18 (9.42)	0.02
**Biochemical data**			
Serum albumin (g/dL)	4.2 ± 0.3	4.2 ± 0.3	0.29

^1^ The subjects were classified into 2 groups according to low-protein diet in all subjects

^2^ The data are presented as means ± standard deviations

^3^T-test was performed to compare parameters according to low-protein diet

^4^ The data are presented as number (percentage)

^5^ Malnourished patients were assessed using modified SGA (subjective global assessment)

^6^ Chi-square test was performed to compare the categorical parameters (malnutrition) according to low-protein diet

## Discussion

This study is a cross-sectional and observational study, in which major foods contributing to the intake of protein, energy, and minerals, DII, and nutritional status of pre-dialysis CKD patients were analyzed to understand differences according to DM comorbidity or disease progression. The contributions of whole grains and legumes to the protein intake were higher in DM-CKD group compared with the Non-DM-CKD group while contribution from the refined grains was lower in the DM-CKD group. Among the foods, intake of whole grains and legumes was higher in the DM-Early-CKD group than in the Non-DM-Early-CKD group. The potential for inflammation in the diet did not differ among the groups in this study. Based on the SGA classification, there were more malnourished subjects in Late-CKD group. Percetange of malnoursihed subjects was lowest in the Non-DM-Early-CKD group and highest in the DM-Late-CKD group. Based on the BIA data, DM-CKD group had lower phase angle compared with Non-DM-CKD group, and Late-CKD group had lower phase angle than the Early-CKD group.

Malnutrition can be screened using serum albumin levels, SGA scores by dietitian’s interview, BIA data, and other methods. Until now, a unified tool has not been used as a standardized nutritional assessment tool. The laboratory markers and physical examination results can be used as complementary tools to each other [19]. Malnutrition status varied in this study depending on the assessment tools used and this is likely due to the difference in components for each assessment tools. In addition, assessment methods might have been not sensitive enough to distingush the difference in these subjects who were not severely malnourished. In order to identify the patients at risk and delay disease progression in pre-dialysis patients, development of more specific tools for CKD patient at the early stage of disease might be needed. Among 256 CKD patients, 31 were malnourished and all of them were moderately malnourished according to the classification based on SGA. The correlation between the BIA and SGA scores of malnourished subjects indicated that better nutritional status was associated with higher PhA and lower ECF (Supplement table 8). The BIA results of pre-dialysis patients reflect the change in nutritional status of CKD patients compared to normal people [20]. Change in cell membrane balance and body fluid balance measured with the BIA can also indicate malnutrition. Under poor nutritional conditions, PhA is reduced and the extracellular fluid is elevated. A low PhA level results from a reduction in body cell mass [21]. Body cell mass is reduced when the body is in a catabolic state [22]. According to Bellizzi et al. [20], the PhA differed between CKD patients and normal people, but there was no difference according to CKD progression stage. ECF is the ratio of extracellular water to total body water. All groups except the Non-DM-Early-CKD group had excess ECF above 0.39. Excess ECF in CKD patients is associated with increased risk of coronary calcification [23]. BMI was higher in the DM-CKD group than the Non-DM-CKD group for both men and women. Visceral fat area, percent body fat and fat mass were higher in the DM-CKD group than Non-DM-CKD group in men. The average BMI of the DM-CKD group was above 25 kg/m^2^ (criteria for obesity) in both men and women. Weight reduction could be beneficial for those with BMI above 25 kg/m^2^, as obesity can lead to structural changes and rapid reduction of kidney function. The effects of weight loss include reduced glomerular hyper-filtration and improved insulin sensitivity [24]. In particular, male CKD patients with DM should make an effort to reduce visceral fat and percent body fat because these are risk factors for CVD [25].

Although protein intake level was not significantly different in men and tended to be different in women depending on DM comorbidity, several foods contributing to the protein intake were significantly different between DM-CKD and Non-DM-CKD groups. The percentage contributions by refined grains, legumes and whole grains to protein intake differed among four groups. The DM-CKD group had higher percent contributions from whole grains and legumes and a lower percent contribution from refined grains compared to the Non-DM-CKD group. Grains are not generally included in the protein source and plant protein sources have lower protein content than animal protein sources [26]. However, consumption of grains and contribution of carbohydrates to energy intake are higher in Asian countries including South Korea compared with US [27]. Grains become an important source of the protein in Korea due to the amount of grains consumed, and the choice of grains (refined vs whole grain) can have a significant impact on the phosphorus and potassium intake levels. Along with the percentage of whole grains and legumes contributing to protein intake, there was also a difference in amount of actual intake between people with and without diabetes. The differences in percent contributions and amount of food intake according to DM comorbidity seemed to be due to the DM patients being older and them choosing whole grains instead of refined grains for the beneficial effects on blood glucose control. KDOQI (Kidney Disease Outcomes Quality Initiative) guidelines recommend a managing target of hemoglobin A1c of ~ 7.0% to delay or prevent the microvascular complications of DM [28]. Whole grains are reported to help the control of blood glucose and insulin homeostasis for healthy subjects [29]. An intervention study showed that the levels of proteinuria emission and C-reactive protein decreased among patients with diabetic nephropathy in the intervention group, in which control group’s protein intake was 70% from animal and 30% from vegetable sources, while intervention group’s protein intake was 35% from animal, 35% from legumes, and 30% from vegetable sources [30]. However, in order to control phosphorus and potassium levels, it should be considered that whole grains contain substantial amount of minerals. Whole grains were the fifth highest contributor to phosphorus consumption and ninth highest contributor to potassium consumption in all subjects among 30 groupings of food used in this study (Supplement table 1). Especially, whole grains contributed more to phosphorus and potassium intake in the DM-CKD group (4th for phosphorus and 6th for potassium intake) (Supplement table 2).

Phosphorus and potassium intakes need be controlled depending on kidney function. 87.1% of all patients were taking ACE (angiotensin-converting enzyme) inhibitors or ARBs (angiotensin receptor blockers) to control high blood pressure. Patients taking these medications need to limit their potassium intake to maintain serum potassium levels within normal range [31, 32]. In this study, antihypertensive medications were used in 223 subjects (87.1%), hyperphosphatemia was present in 22 subjects (8.6%) and hyperkalemia was present in 28 subjects (10.9%). Hyperphosphatemia and hyperkalemia become more prevalent (19.7 and 28.9%, respectively) among CKD patients in stages 4 and 5. Hyperphosphatemia causes bone-mineral disorders that increase the risk of developing CVD, and hyperkalemia can cause arrhythmia [33]. According to the KDIGO guidelines, sufficient energy intake and a minimum amount of protein intake is recommended for CKD patients [11]. If phosphorus and potassium intakes need to be controlled with the progression of kidney disease, advice regarding consumption of whole grains and legumes should be considered in DM-CKD patients. However, decreased consumption of whole grain should not be achieved by simply decreasing the total intake of whole grains and legumes because this may result in decreased energy and protein intakes.

A lower DII reflects an anti-inflammatory diet. When the DM-Early-CKD, Non-DM-Late-CKD, and DM-Late-CKD groups were compared to the Non-DM-Early-CKD group, these three groups consumed relatively more anti-inflammatory diet but the results were not significant. It has been  shown that a higher DII is associated with increased prevalence of type 2 diabetes mellitus [34] and reduced kidney function [15, 35]. It seems that the lack of significant difference in dietary inflammatory potential among the groups in this study was because patients with progressed disease (patients in the Late-CKD group) and those with diabetes were already making some efforts to manage their diets. Consumption of relatively more pro-inflammatory diet by the Non-DM-Early-CKD group might be related to the age of this group being younger than other three groups. It was reported that high carbohydrates or fat diets are associated with raising the inflammatory response [36]. CKD patients can have a relatively higher percentage of carbohydrates and fat intake as they practice low-protein diets.

This study has several limitations. First, this study is an observational study, which does not identify causal relationships between decreased kidney function and intake of specific food groups or DII. Second, the diagnostic standard for CKD used only serum creatinine without proteinuria. Third, physical activities were not examined in this study. Fourth, number of nutritional education subjects received varied. Average number of nutritional education was 1.8 times but one subject received more than 20 education sessions. Therefore, the degree to which diets were modified could be quite different and the effects of nutrional education or changes in food intake were not analyzed. Despite these limitations, the top 10 food groups among 30 food groups contributing to protein intake were identified and ranked according to the contribution. Registered dietitians suggest limited protein intake for CKD patient, but prior to this study, there has been no basis for determining which foods substantially contribute to protein intake. Also, we have provided detailed information on nutritional status by using BIA and SGA in pre-dialysis patients. This study analyzed the association between kidney disease patients and DII in Asia. In addition, eGFR was calculated using the CKD-EPI equation rather than the MDRD (Modification of Diet in Renal Disease) study equation. The CKD-EPI equation predicts clinical risk more accurately than the MDRD study equation for Asians [37].

## Conclusions

In conclusion, we identified differences in nutritional status and food contributions to protein intake among CKD patients with and without diabetes at different stages of kidney function. The nutritional assessment results varied depending on the BIA, SGA and serum albumin parameters and it is suggested that development of more specific tools that can identify malnutrition among CKD patient at the early stage of disease might be needed. Significant differences in consumption of whole grains and legumes between CKD patients with or without DM were observed. Whole grains were high contributor to protein, phosphorus and potassium intake. This result suggests that advice regarding consumption of whole grains and legumes may be necessary for DM-CKD patients if phosphorus and potassium intake levels need to be controlled. Patients with relatively high protein intakes were found to be in better nutritional status than patients with lower levels of intake. This study helps understanding the characteristics of food sources in the diet of CKD patients and provides some basis for nutritional intervention to help pre-dialysis patients control their nutritional status and food intake depending on the presence of diabetes.

## Supplementary information

**Additional file 1: Supplement table 1.** Major food sources contributing to energy and nutrients intakes in all CKD patients (*n* = 256). **Supplement table 2.** Major food sources contributing to energy and nutrients intakes in DM-CKD groups (*n* = 107). **Supplement table 3.** Major food sources contributing to energy and nutrients intakes in Non-DM-CKD groups (*n* = 149). **Supplement table 4.** Bioelectrical impedance analysis (BIA) measurement of the male subjects according to tertiles of protein intake. **Supplement table 5.** Bioelectrical impedance analysis (BIA) measurement of the female subjects according to tertiles of protein intake. **Supplement table 6.** Bioelectrical impedance analysis (BIA) measurement of the male subjects according to low-protein diet (< 0.8 g/kg/day). **Supplement table 7.** Bioelectrical impedance analysis (BIA) measurement of the female subjects according to low-protein diet (< 0.8 g/kg/day). **Supplement table 8.** Pearson’s correlation coefficients between BIA data and SGA score in malnourished patients.

## Data Availability

The datasets used and/or analysed during the current study are available from the corresponding author on reasonable request.

## Abbreviations

BIA: Bioelectrical impedance analysis
BMI: Body mass index
CKD: Chronic kidney disease
CVD: Cardiovascular disease
DII: Dietary inflammatory index
DM: Diabetes mellitus
ECF: Extracellular fluid
eGFR: Estimated glomerular filtration rate
ESRD: End-stage renal disease
GFR: Glomerular filtration rate
PhA: Phase angle
SGA: Subjective global assessment
CKD-EPI: Chronic Kidney Disease Epidemiology Collaboration
